# Malignant Transformation of Long-Standing Pseudoepitheliomatous Keratotic and Micaceous Balanitis (PKMB) Presenting as Urethral Obstruction

**DOI:** 10.7759/cureus.58671

**Published:** 2024-04-21

**Authors:** Chrysovalantis Gkekas, Ioannis Tsikopoulos, Stamatios Katsimperis, Georgios Antoniadis, Dimitrios Papadopoulos

**Affiliations:** 1 Urology, 404 Military Hospital of Larissa, Larissa, GRC; 2 Urology, Second Department of Urology, National and Kapodistrian University of Athens, Sismanogleio General Hospital, Athens, GRC; 3 Urology, General Hospital of Larissa, Larissa, GRC; 4 Urology, West Middlesex University Hospital, Isleworth, GBR

**Keywords:** glansectomy, urology and oncology, glans penis, malignancy surgery, pseudoepitheliomatous keratotic and micaceous balanitis

## Abstract

Hereby, we present a rare case of malignant transformation in a long-standing case of pseudoepitheliomatous keratotic and micaceous balanitis (PKMB), which typically affects older men. PKMB presents as whitish or silvery keratotic plaques on the glans and can remain stable for years, leading to potential confusion regarding its progression. The patient in this case experienced urinary obstruction due to tumorous ingrowth, prompting an investigation. Despite previous treatments, including fluorouracil (5-FU) and cryoablation, the lesion persisted, eventually growing in size and becoming malodorous. Initial biopsy showed PKMB without malignancy, but subsequent deeper biopsy revealed verrucous carcinoma. The patient underwent glansectomy and reconstruction with a full-thickness skin graft, achieving a disease-free state postoperatively. The paper underscores the importance of thorough investigation for malignancy in PKMB cases, the possibility of deeper malignancy missed by superficial biopsy, and the need for early diagnosis to enable organ-sparing treatments.

## Introduction

Pseudoepitheliomatous, keratotic, and micaceous balanitis (PKMB), or balanitis of Civatte, is a predominantly coronal balanitis arising from the sulcus area but can extend to the tip of the glans and the subcoronal preputial epithelium. The condition's name is attributed to the formation of mica-resembling plaques that can potentially build up into forming horns. It was initially described in 1961 by Lortat-Jacob et al. [1]. Histology typically demonstrates acanthosis, hyperkeratosis, and pseudoepitheliomatous hyperplasia with no cytological atypia, although malignant transformation has been consistently described along its course. A periodic acid-Schiff (PAS) stain is useful to exclude a fungal infection [2]. Biopsy is almost always required to exclude malignancy.

PKMB is usually encountered in the senile male population and predominantly in those circumcised. It is a slow-growing, non-infectious disease; nonetheless, it can be confounded with superimposed infections and, due to its protracted course, it is common for the average patient to undergo repeated cycles of topical treatment. It manifests as a hyperkeratotic plaque on the penile glans, with overlying thick micaceous flakes [3]. Oftentimes, mica scales can deteriorate to the point of forming keratotic horns. The condition is generally asymptomatic but, not uncommonly, associated fissuring, maceration, and ulceration can cause irritation and discomfort. The exact pathophysiology of PKMB remains unclear. The clinical course of PKMB is chronic and associated with frequent recurrences after treatment [2]. The average patient seeks medical advice to treat a growing, unsightly lesion that can be complicated with infection, causing local pain, itching, and odorous discharge. In our case, it is impressive the fact that urinary obstruction was the principal complaint caused by tumorous ingrowth and ultimately compression of the urethra.

The patient was not initially alerted by the change in the lesion’s appearance. It had happened with his lesion in the past to be irritated, moist, and producing discharge but that was due to friction or scratching and subsided after local antimicrobial treatment.

## Case presentation

**Figure 1:**
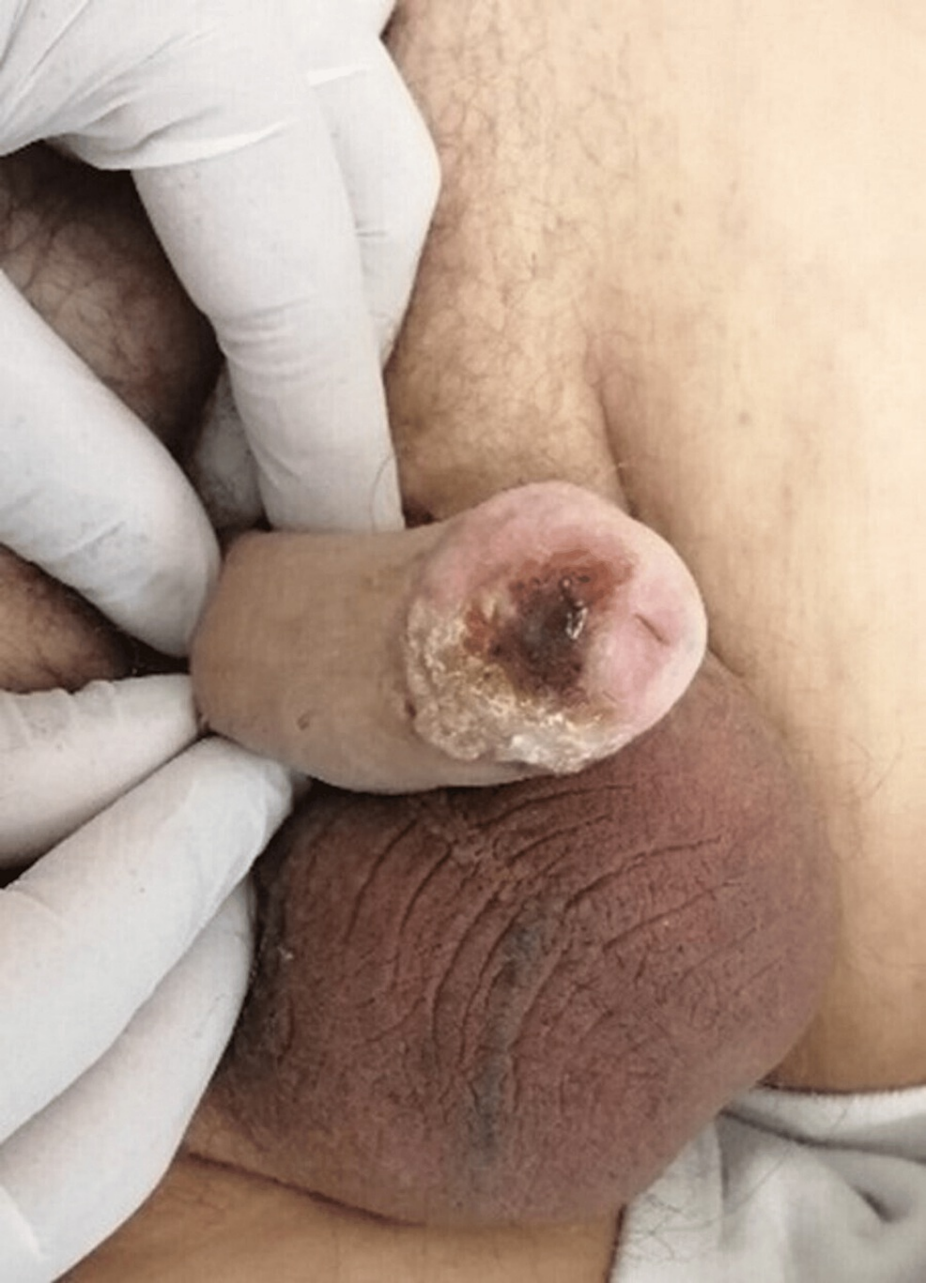
Clinical picture describing the appearance of the lesion when stable and asymptomatic

A 69-year-old patient presented to the urology outpatient clinic with a flat, hyperkeratotic, and dry lesion on the penile glans. The patient was circumcised and reported that the lesion was first noticed a couple of months after his circumcision, 22 years ago. It had remained stable in size for many years, with only occasional sanguineous oozing from its surface, which he attributed to irritation after intercourse (Figure 1). These symptoms drove him to dermatology and urology clinics from time to time, where he had been offered fluorouracil (5-FU) treatments twice, cryoablation, and a punch biopsy. Histology at the time showed PKMB, but the subsequent treatments had overall failed to make the lesion disappear; luckily, it remained unchanged and stable for many years.

His complaint at presentation was of persistent odorous discharge from the lesion, which itself grew in size and could not be calmed with soaps and emollient creams as it used to in the past. The patient asked for antibiotics, based on his previous experience, unaware of the nature of his disease.

**Figure 2:**
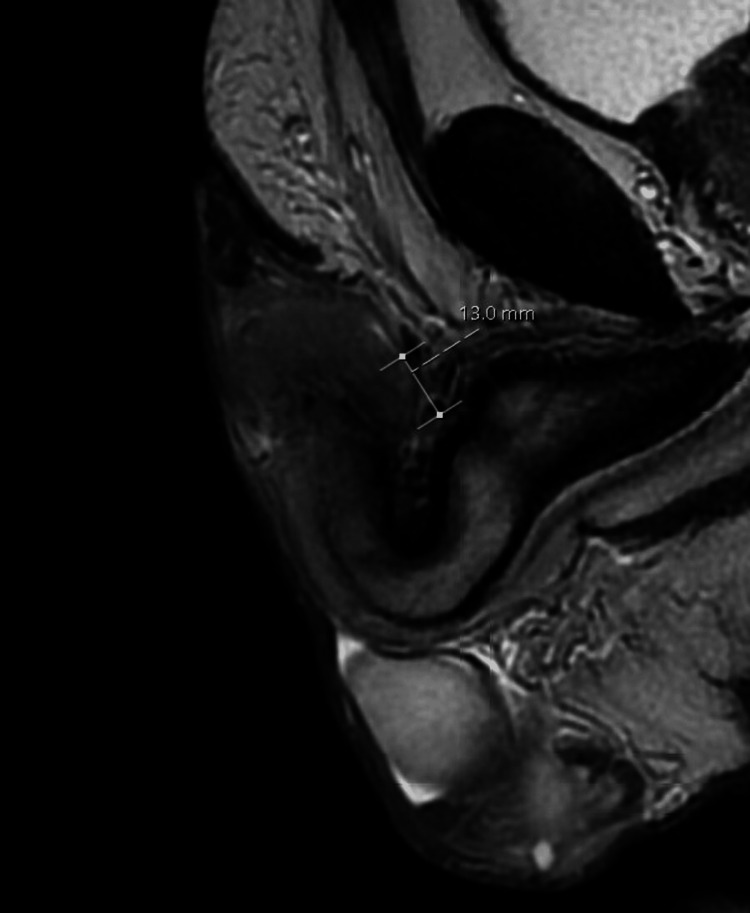
Sagittal MRI plane (T2-weighted image) showing the lesion, measuring 13 mm

Physical examination revealed a fleshy, flat lesion with scales, involving the right half of his glans and the adjacent preputial skin along his circumcision scar. The surface of the lesion appeared moist and when compressed, it drained malodorous discharge. Upon palpation, the lesion gave the impression it extended deep into the glans, resembling a hard nodule. The urethral meatus was seemingly unaffected and the navicular urethral patency was confirmed with an 18Fr catheter that was easily introduced, although an area of resistance could be felt halfway through its length. The patient reported no difficulty in passing urine at the time. He had no palpable inguinal nodes. A swab from the pus was sent for culture, which grew Pseudomonas spp, and antibiotics were commenced as per sensitivity results. Additional investigations with an MRI of his penis revealed a 1.2 cm lesion confined within the glans with no urethral or cavernosal infiltration and no lymphadenopathy (Figure 2). After an in-depth consultation, he agreed to be investigated further with a repeat biopsy. A shave biopsy revealed PKMB without any finding indicative of malignancy.

**Figure 3:**
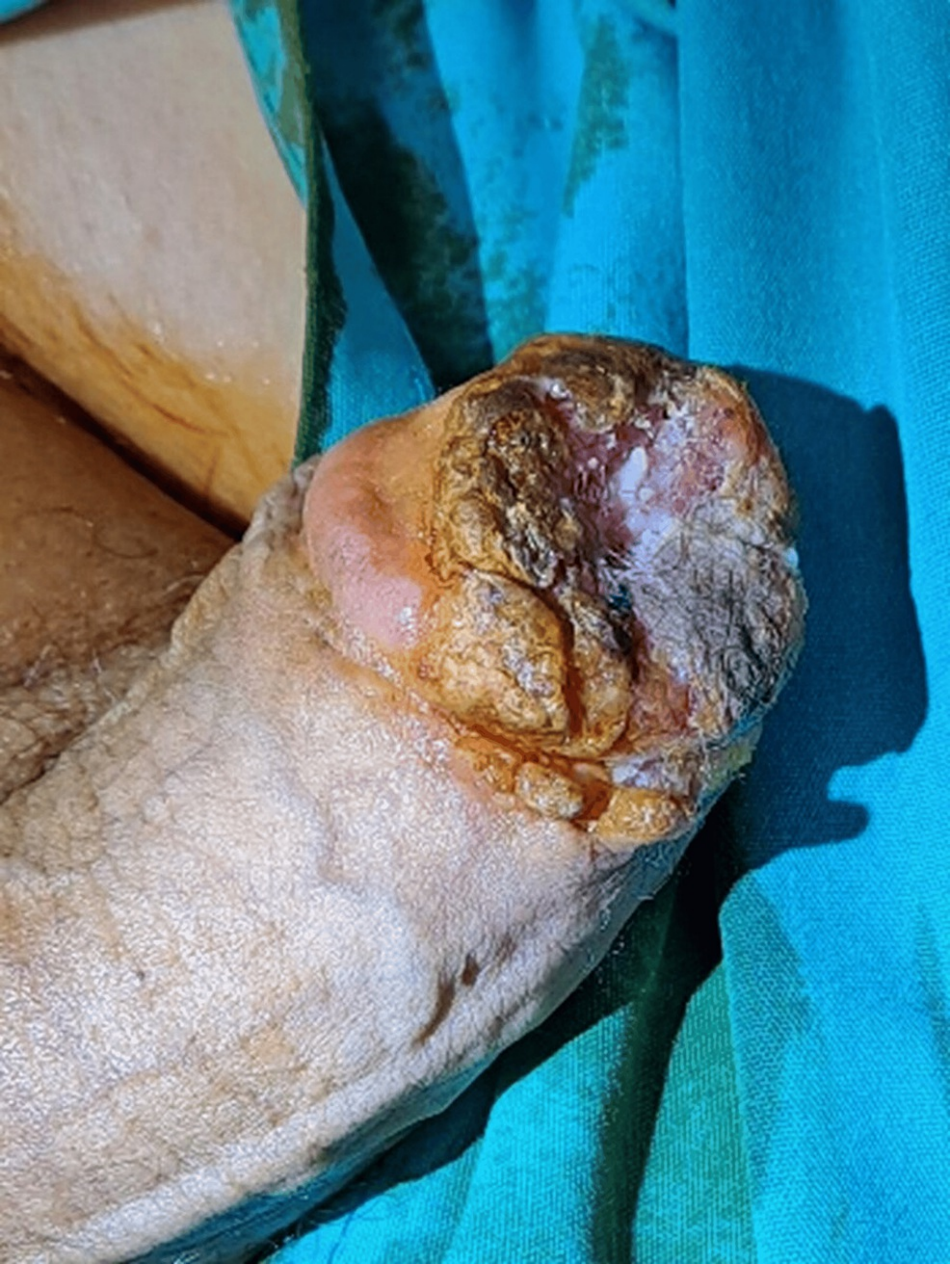
Progressive disease after the wedge biopsy with foci cancerous cells

A telephone follow-up three weeks following his biopsy found him improved and satisfied with his progress, and a face-to-face appointment was scheduled for two months later. Due to the COVID outbreak and the constraints on clinical appointment lists, the patient's visit to the clinic was delayed, and 10 months later, he experienced intense difficulty in passing urine, which led him to the emergency department. It was then apparent that the lesion had considerably grown in size, and the urethral meatus was now involved. The patient could only pass drops of urine from a barely visible urethral opening; therefore, a suprapubic catheter (SPC) was placed, and the patient was scheduled for surgery. He declined the proposed immediate glansectomy and opted for delayed surgery and repeat investigation against our advice. He underwent a repeat MRI, which showed a 1.8 cm penile mass abutting and compressing the navicular urethra with inconspicuous infiltration of the right corporal tip. Still, no associated lymphadenopathy was noticed. The patient was reluctant to undergo radical surgery even after the MRI results, but he agreed to a deeper, wedge biopsy, which now revealed limited focal atypia indicative of verrucous carcinoma (Figure 3).

**Figure 4:**
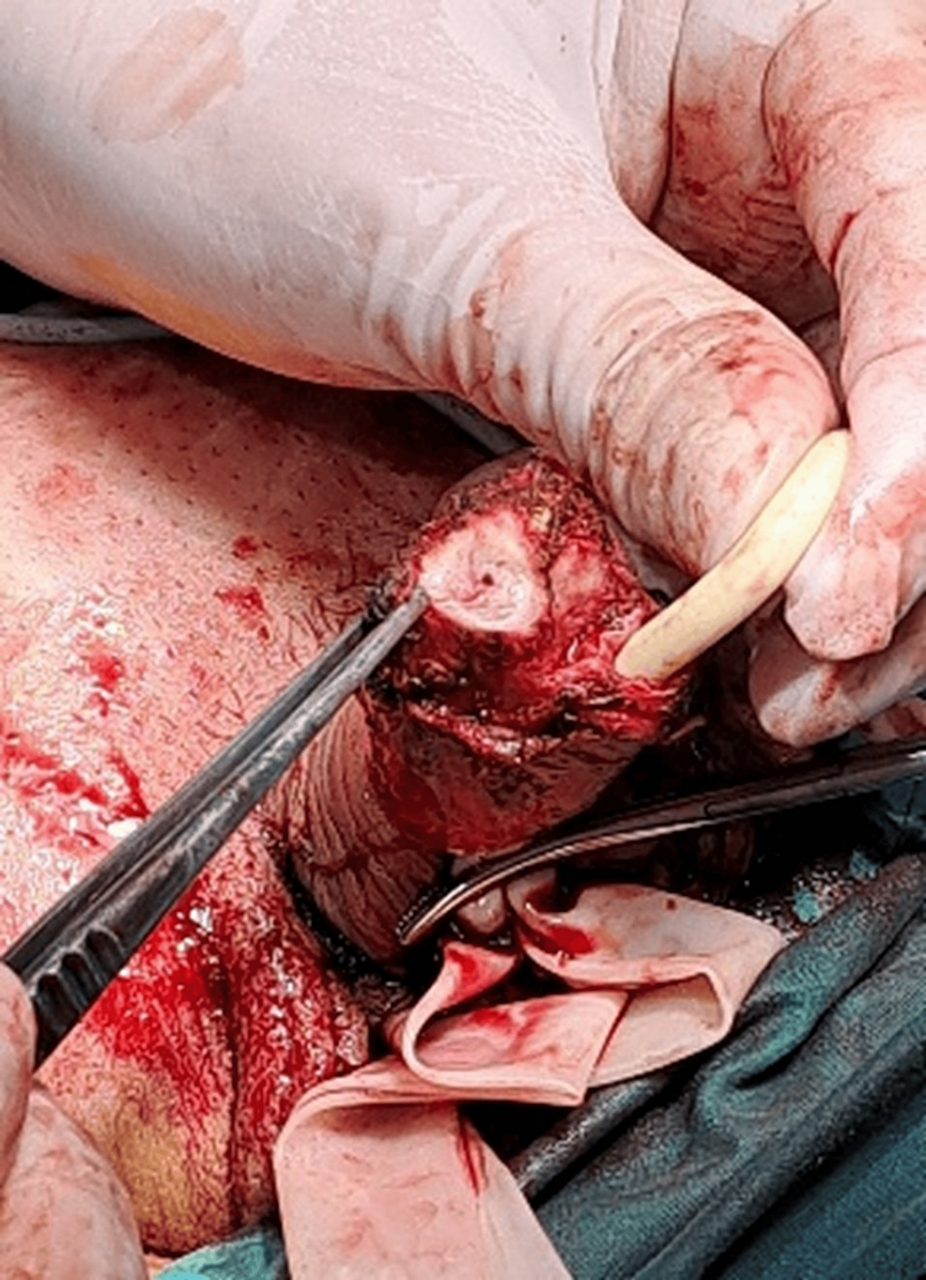
Intraoperative clinical image of glansectomy and excision of the right corporal tip

**Figure 5:**
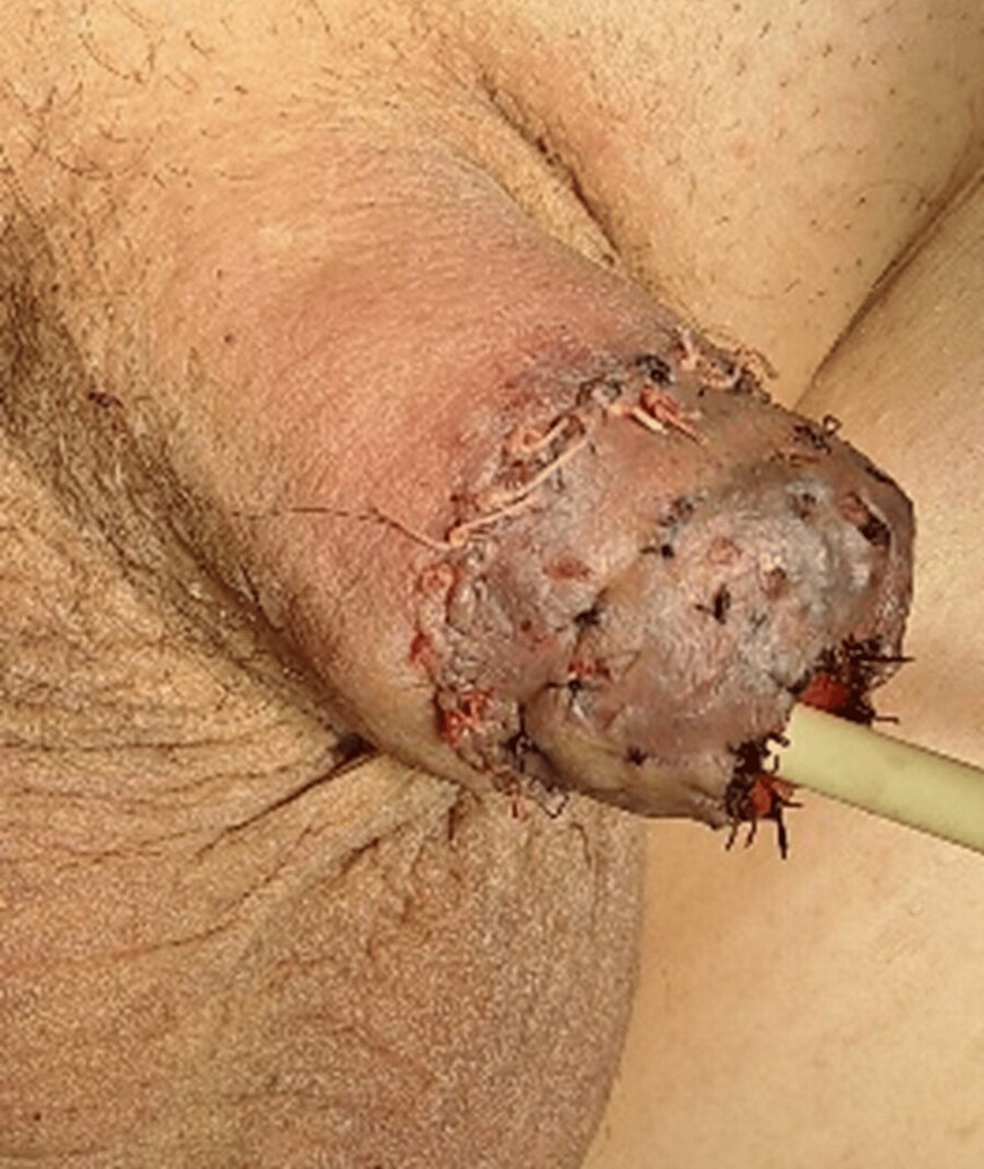
Image of the full thickness skin graft quilted in place

He was eventually managed with glansectomy, resection of the right corporal tip, and full thickness skin graft (FTSG) pseudo-glans reconstruction (Figures 4, 5).

**Figure 6:**
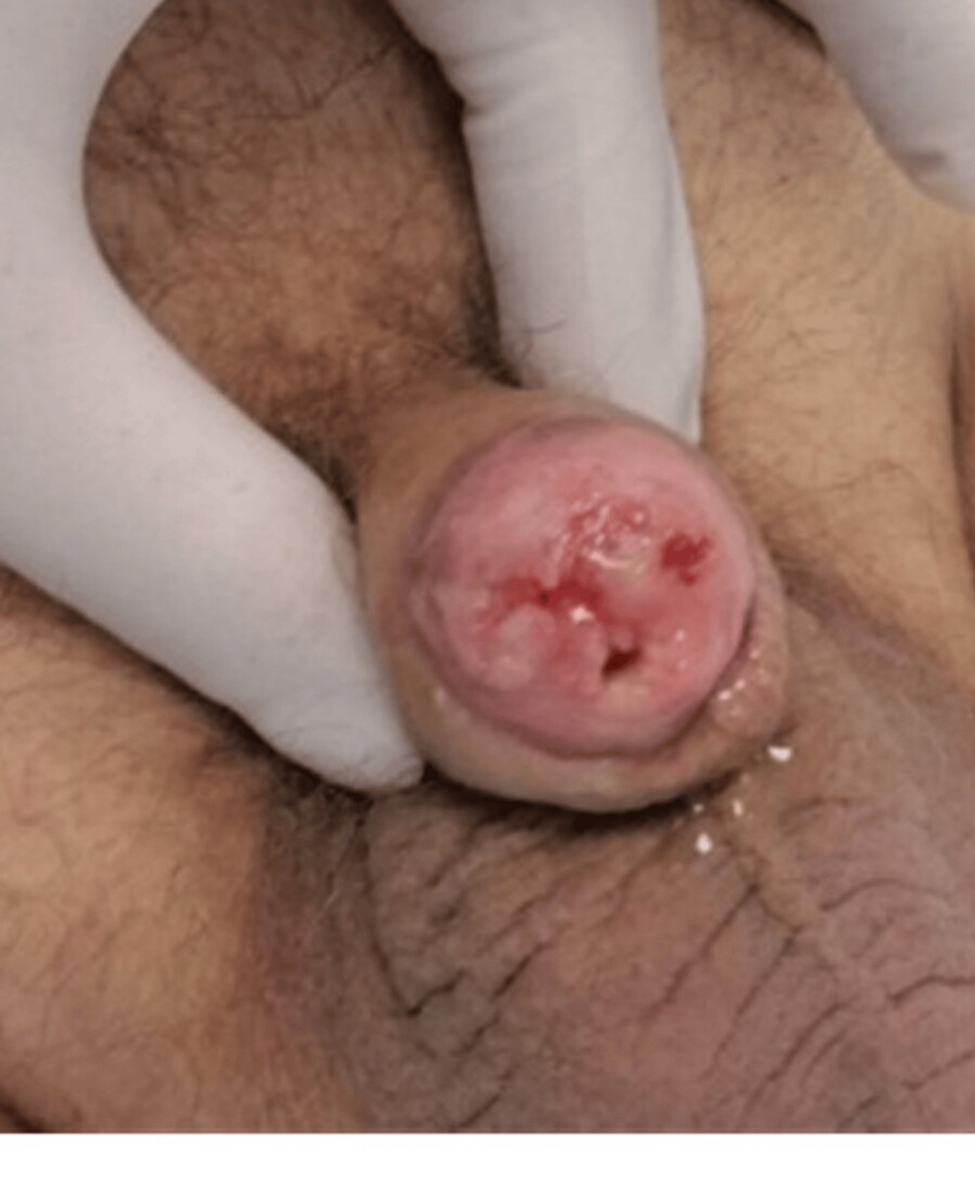
Pseudoglans’ appearance at four months, patulous meatus

Ipsilateral ultrasound-guided fine-needle aspiration cytology (FNAC) of a sonographically suspicious lymph node, at two weeks postoperatively, failed to reveal involvement. After the focal loss of the graft, just above the resected right corporal tip, which was managed conservatively, the overall take was successful and he remains disease-free five months postop with an acceptable aesthetic result and a patulous meatus (Figure 6). 

## Discussion

PKMB represents a rare skin condition affecting the glans' epithelium and frequently the adjacent preputial skin. Almost invariably, the preputial skin is concomitantly affected along with the glans, and due to the scarcity of data, it is unknown whether isolated preputial lesions can exist without the glans' involvement. The condition typically affects older men with a mean age at presentation above 60 years and mostly those uncircumcised. Macroscopically, it manifests as whitish or silvery keratotic plaques or even horns, resembling mica minerals on the glans, hence the name. The course of the disease is very slow and can remain stable for years, but in ominous cases, it can be complicated with verrucous or squamous cell carcinomas. Although once considered entirely benign and extremely rare, accumulating reports have shed light on its malignant propensity [3,4]. The clinical course of PKMB is chronic and associated with recurrences after treatment [5]. In isolated cases managed with local agents like 5-FU, full recovery has been reported [6,7]. This slow progression confuses the patient and the clinicians, missing the early signs of malignant transformation. In our case, malignant transformation was diagnosed 22 years after onset due to urinary obstruction of the distal urethra. There was an apparent delay in the diagnosis leading to more invasive treatment, which would have been avoided had a higher degree of suspicion existed.

Spencer et al., in a case series study, presented findings indicating that PEKMB resembles chronic, often undiagnosed or misdiagnosed, and treatment-resistant lichen sclerosus. Early diagnosis and treatment are essential to reduce the risk of squamous carcinogenesis. Glans resurfacing and split skin graft reconstruction show promise as effective treatments for refractory cases [8].

## Conclusions

In conclusion, the diagnosis of pseudoepitheliomatous keratotic and micaceous balanitis (PKMB) should prompt a comprehensive investigation into potential concurrent penile cancer. The findings underscore the limitations of superficial biopsies in detecting deeper foci of malignancy, emphasizing the need for thorough examination and consideration of deeper tissue sampling. Furthermore, the case highlights the insidious nature of PKMB, as it can evolve into cancer even after a prolonged latency period, necessitating vigilance and regular follow-up. Early detection is paramount for enabling organ-sparing treatments and improving patient outcomes. Clinicians should have a high index of suspicion when it comes to those lesions. Additionally, PKMB that proves resistant to topical therapies should prompt consideration of excision to prevent further progression and mitigate the risk of malignant transformation. These key points underscore the importance of proactive management strategies in addressing PKMB and its potential sequelae, ultimately guiding clinical practice for optimal patient care.
